# Economic evaluation of a person-centred care intervention with a digital platform and structured telephone support for people with chronic heart failure and/or chronic obstructive pulmonary disease: results from a randomised controlled trial in Sweden

**DOI:** 10.1136/bmjopen-2024-093083

**Published:** 2025-10-09

**Authors:** Benjamin P Harvey, Emmelie Barenfeld, Andreas Fors, Inger Ekman, Karl Swedberg, Hanna Gyllensten

**Affiliations:** 1 University of Gothenburg Institute of Health and Care Sciences, Gothenburg, Sweden; 2 University of Gothenburg Centre for Person-Centred Care, Gothenburg, Sweden; 3 Department of Health and Rehabilitation, University of Gothenburg Institute of Neuroscience and Physiology, Gothenburg, Sweden; 4 University of Gothenburg Centre for Person-Centred Care, Goteborg, Sweden; 5 University of Gothenburg Institute of Health and Care Sciences, Goteborg, Sweden; 6 Department of Molecular and Clinical Medicine, Sahlgrenska Academy, Goteborg, Västra Götaland, Sweden; 7 University of Gothenburg Centre for Person-Centred Care, Goteborg, Västra Götaland, Sweden; 8 Institute of Health and Care Sciences, University of Gothenburg, Goteborg, Sweden

**Keywords:** Patient-Centered Care, Person-Centered Care, Heart failure, Pulmonary Disease, HEALTH ECONOMICS, eHealth

## Abstract

**Objectives:**

The aim of the study was to evaluate the healthcare costs and effects of a remote person-centred care (PCC) add-on intervention compared with usual care for people with chronic heart failure (CHF) and/or chronic obstructive pulmonary Disease (COPD) from a societal perspective.

**Design:**

A cost-effectiveness analysis (CEA) based on the results from a randomised controlled trial.

**Setting:**

The study was conducted from August 2017 until June 2021 within nine primary care centres across Western Sweden.

**Participants:**

Participants in the study had a diagnosis of COPD (J43.0, J44.0–J44.9) and/or CHF (I50.0–I50.9).

224 patients were randomly allocated to the study groups. After two withdrawals, the final intention-to-treat analysis included 110 participants in the intervention group and 112 in the control group.

**Interventions:**

Both the intervention and control group received usual care through their primary care centres. In addition, the intervention group participated in a remote PCC add-on intervention consisting of a digital platform and structured telephone support.

**Primary outcome:**

Incremental cost-effectiveness ratio using direct healthcare costs, productivity loss and prescription drug costs, compared with health effects measured using the EuroQoL questionnaire (EQ-5D-3L) over a 2-year time horizon.

**Results:**

The intervention group had lower healthcare utilisation in inpatient care, specialised outpatient care and reduced productivity loss. The CEA showed incremental effects of 0.0469 quality-adjusted life years and incremental costs of SEK −68 533 (Swedish crowns). The PCC alternative was both more effective and resulted in lower healthcare costs compared with usual care, that is, PCC was dominant.

**Conclusions:**

The results of this CEA demonstrated that a remote PCC add-on intervention for people with COPD and/or CHF had lower healthcare costs and higher health-related quality of life compared with usual care.

**Trial registration number:**

NCT03183817 ClinicalTrials.gov.

**STRENGTHS AND LIMITATIONS OF THIS STUDY**

- A randomised controlled trial design reduced the risk of bias, ensuring between group differences were based solely on the intervention of interest.
- Near-complete register data at both the national and regional levels provided detailed overviews of costs and healthcare utilisation.
- Costs were reported using a societal perspective and presented descriptively from a healthcare and patient perspective, respectively.
- The intervention group reported almost twice as many missing EQ-5D-3L values at 3, 6, 12 and 24 months compared with the control group.
- Primary care costs were estimated from regional guidelines that are based on weighted contacts.

## Introduction

 Chronic heart failure (CHF) and chronic obstructive pulmonary disease (COPD) are among the world’s leading causes of morbidity and mortality.^1^ Shared pathophysiological characteristics and common risk factors, such as smoking and increased age, mean that these two diseases often co-exist, increasing the risk of mortality. Both diseases are similarly impactful on patients’ daily lives, with studies^2 3^ highlighting comparable levels of self-efficacy, health status, dyspnoea, fatigue, pain and anxiety. The economic burden of these diseases also poses significant societal challenges, with the USA spending an estimated $31 billion treating CHF in 2012, costs that are predicted to increase to upwards of $70 billion by the year 2030.^4^ The total annual costs of COPD increased from $37 billion to $50 billion from 2004 to 2010 in the USA, with continental Europe experiencing comparable expenditure patterns.^5^ The burden of these diseases highlights a need to innovate on current methods for providing healthcare that promote patient involvement to contain costs and facilitate a transition to more sustainable healthcare systems.

In addressing financial sustainability within healthcare, fundamental concepts of patient engagement and shared decision-making are being promoted through the practice of person-centred care (PCC).^6^ This requires healthcare professionals’ (HCPs) responsiveness to patients’ expressed abilities, needs, concerns and expectations, as well as their subjective understanding of living with illness. Operationalising PCC has demonstrated several economic benefits, contributing to reduced healthcare utilisation, productivity loss and prescription drug use, while also having positive economic effects for family, friends and unpaid carers.^7^ In further attempts to restructure and optimise the delivery of care, services are transitioning to digital communication and information technologies (eHealth). Providing PCC through an eHealth medium has demonstrated positive results with regard to self-management; however, the cost-effectiveness of these interventions is less well studied.^8^ Therefore, the aim of the study was to evaluate the cost-effectiveness of a remote PCC add-on intervention compared with usual care for people with CHF and/or COPD from a societal perspective.

## Methods

A cost-effectiveness analysis was performed using data from the PeRsOn-cenTrEd Care at disTance (PROTECT) randomised controlled trial (NCT03183817), a remote PCC add-on intervention combining a digital platform and structured telephone support for people with COPD and/or CHF.^8^ The reporting was performed in accordance with the Consolidated Health Economic Evaluation Reporting Standards criteria^9^ and the Consolidated Standards of Reporting Trials guidelines.^10^ The data were analysed from a societal perspective with a 2-year time horizon. A healthcare payer perspective and patient perspective were also explored to highlight the potential impact of the cost components for the different actors involved in the participation and delivery of healthcare.

### Participants

**Figure 1:**
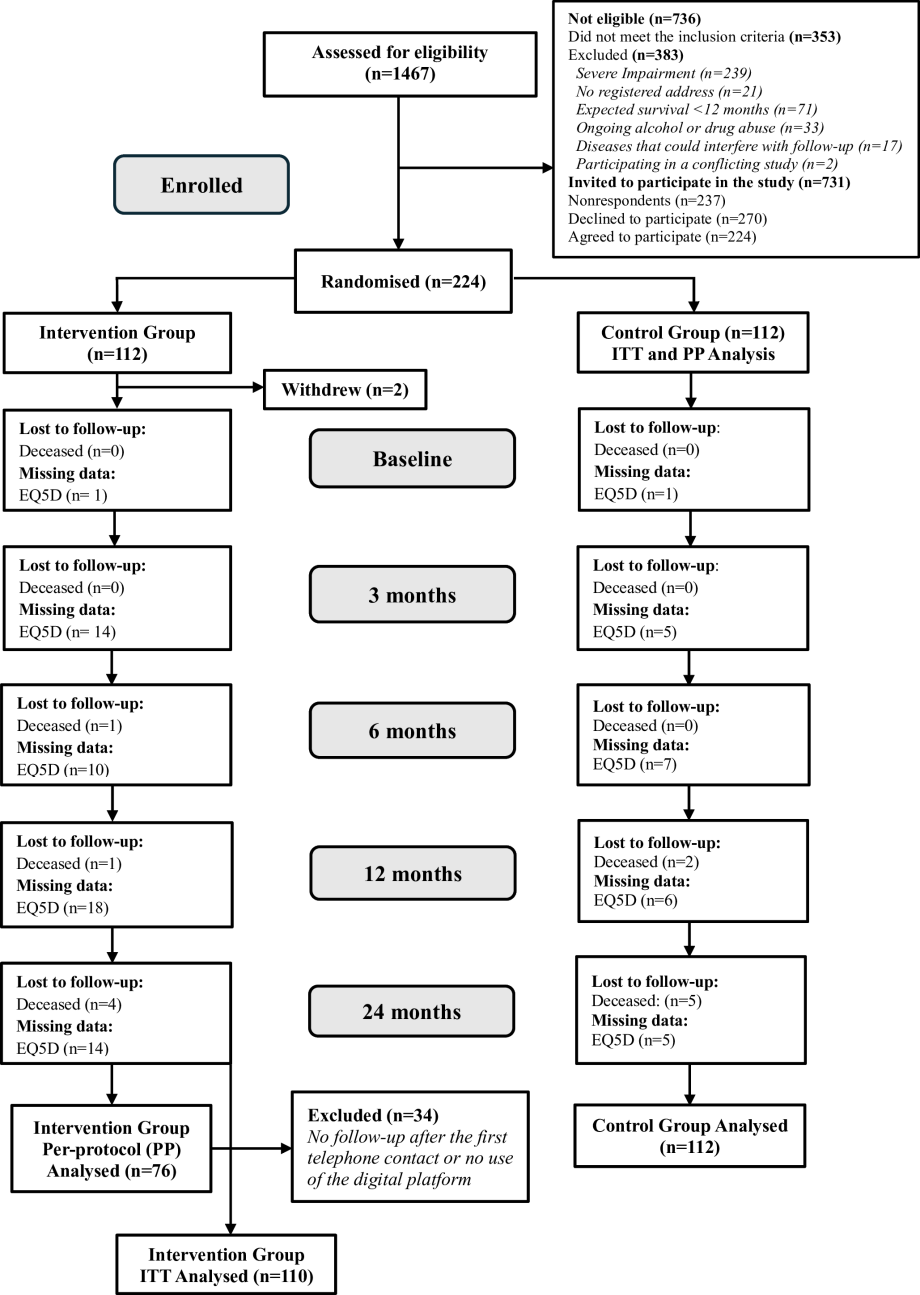
CONSORT flow chart. EQ-5D-3L, health-related quality of life measured by the Euroqol 5 dimensions health state questionnaire; ITT, intention-to-treat: PP, per protocol.

Participants in the study had a diagnosis of COPD (J43.0, J44.0-J44.9) and/or CHF (I50.0-I50.9) assigned by a general practitioner or an internist in medicine or cardiology, were registered at one of the nine primary care clinics, understood written and spoken Swedish and had access to a digital device with internet connectivity (figure 1). The exclusion criteria were: (1) severe impairment that hindered the use of a digital device, preventing participation in the eHealth support; (2) no registered address; 3) expected survival of <12 months; (4) ongoing, documented diagnosis of alcohol or drug abuse; (5) diseases that could potentially interfere with the planned follow-ups; and (6) participation in any conflicting studies.

In order to achieve 80% power on a p value of 0.05, a required sample size of 91 patients in each group was calculated to achieve a 20% to 40% improvement in the composite primary endpoint, changes in general self-efficacy, hospitalisation or death 6 months after randomisation.^11^ Accounting for attrition, approximately 110 participants were originally planned for the intervention and control group. Recruitment of the participants took place between August 2017 and June 2019.^8^ In total, 1467 people were assessed for eligibility, with 736 patients not meeting the inclusion/exclusion criteria after the initial screening of medical records. The remaining 731 patients were deemed eligible and posted an information letter inviting them to contact an HCP for further information. Participants who did not contact an HCP after a 2-week period were contacted via phone and asked to participate. A total of 224 patients consented to participating in the trial after accounting for non-respondents (n=237) and those that declined to participate (n=270). Further details on reasons for not fulfilling the eligibility criteria and description of the recruitment process have been published previously.^8^

A total of 224 patients were recruited and randomly allocated to the study groups; however, after two withdrawals, the final intention-to-treat (ITT) analysis included 110 participants in the intervention group and 112 in the control group. Randomisation was conducted applying a computer-generated list created by a third party and had a 1:1 allocation ratio and was stratified by age (<65 or ≥ 65) and diagnosis (CHF, COPD, or both CHF and COPD).

### Remote person-centred care (PCC) add-on intervention and usual care

Both the intervention and control group received usual care through their local primary care and specialised care centres in accordance with current medical guidelines. Additionally, the intervention group was provided with a remote PCC add-on intervention for 6 months, a digital platform and structured telephone support. The digital platform was designed and developed by the University of Gothenburg’s Centre for PCC to enable the operationalisation of person-centredness with respect to the Gothenburg Model.^12^ Partnership was emphasised through collaboration and mutual respect and is the most important concept in the model as it allows patients to use their resources and capacities, taking responsibility for their care in collaboration with HCPs. Therefore, the patient and HCP discussed and agreed on common goals in the personal health plan; these goals were followed up by both parties.

First, a health plan was co-created via telephone conversation with an allocated HCP that encapsulated the patient’s goals, resources and needs, derived from the patient’s narrative. The pathways to achieving the set goals, resources for self-care and support needed were also documented.^1^,^7^ The digital platform facilitated two-way communication between the patient and HCP, utilising private messages and providing access to the documented health plan, self-rated health scores and other healthcare resources (eg, links to disease specific information, support centres). This contributed to increasing transparency, ensuring that both the HCP and patient had access to the same information on current health status and planned actions. Both the patient and the HCP had access to the digital platform, with the patient invited to customise access, allowing for informal carers, family or friends to view the information. Follow-up meetings via telephone provided opportunities to revise and modify the health plan when applicable in accordance with any changes in the patient’s needs or goals. The key differences in the care provided between the intervention and control group were that in the control group, participants did not co-create a health plan with an HCP, receive follow-up phone conversations or have access to the digital platform. Both the intervention and usual care can include some level of remote encounters and asynchronous contacts; however, the intervention included no face-to-face contacts. A more detailed description of the intervention has been published elsewhere.^8^

### Data collection

Data collection was conducted over a 2-year period from randomisation of each participant. Patient characteristics, demographics and diagnoses were retrieved from medical records. Postal questionnaires collected patient reported measures, including health-related quality of life (HRQoL), at baseline, 3, 6, 12 and 24 months after randomisation. If needed, data were collected by phone.

 Patient-level data were collected from several patient registers; (1) socioeconomic variables (Longitudinal Integrated Database for Health Insurance and Labour Market Studies, held by Statistics Sweden); (2) utilisation of primary care, specialised outpatient care, inpatient care, polyclinical care (ie, inpatient day care less than 24 hours) and diagnosis-related groups (DRGs) were provided by Region Västra Götaland’s regional patient register VEGA; (3) amounts and costs of dispensed pharmaceuticals (Swedish Prescribed Drug Register, the National Board of Health and Welfare); (4) cause and time of death (Cause of Death Register, the National Board of Health and Welfare); and (5) compensation for productivity loss due to disability and/or illness (from the Micro Data for the Analysis of Social Insurance, MiDAS, database, held by the Swedish Social Insurance Agency).

 Time allocated to the intervention for both the participants and HCPs was collected using a project specific communications list (eg, phone calls, support, health plan follow-ups), manually from the digital platform (eg, messages, documented health plans), and a process evaluation questionnaire sent out to the participants 6 months after randomisation.

### Health-related quality of life

The EQ-5D-3L (EQ-5D) was used to calculate quality-adjusted life years (QALYs).^13^ The EQ-5D has five dimensions: mobility, self-care, usual activities, pain/discomfort and anxiety/depression. Within each dimension, the respondent can choose from one of three health states: (1) no problems, (2) some problems or (3) severe problems. The Swedish experience-based value set^14^ provided utility weights to QALY calculations for the cost-effectiveness analysis. At any measurement point, a missing response was handled by applying multiple imputation. A detailed description of the imputation process can be found in online supplemental material 1).

### Healthcare costs

Unit costs for primary care were calculated using the average unit price estimations recommended by the Swedish Association of Local Authorities and Region’s Statistics for Healthcare and Regional Development, 2021.^15^ All costs were calculated with respect to the performing HCP (physician or other HCP, ie, registered nurse, occupational therapist, physiotherapist etc.). Other HCP and group visits were priced at 40% the cost of a consultation with a physician. Team and group/team visits, where a patient meets a ‘care team’, were defined as utilising more than one HCP and priced at one and a half times the cost of a consultation.^16^ Care provided in the home was calculated at two times the unit cost of a consultation. Indirect care (telephone consultation) was priced at one-third of the unit cost of a consultation.

Per-patient total healthcare costs for inpatient care, polyclinical care and specialised outpatient care were calculated using the 2017–2021 DRG weights, times the average cost per healthcare visit in 2021.^17^ Missing DRG weights for specialised outpatient care were calculated using the same method applied to primary care, however, using the average cost of a specialist consultation in 2021.^15^ Polyclinical care and inpatient care had complete DRG information and therefore no additional imputations were required. Healthcare utilisation was calculated using 2021 prices and therefore not inflation adjusted. Amounts and costs of dispensed pharmaceuticals were calculated using the total cost excluding taxes. The direct costs for HCPs’ salaries were calculated using the mean hourly wage and social contributions (personal communication, Glenn Sandvik 2023-06-07), multiplied by the total hours spent in patient-facing meetings, documentation and communication as part of the intervention.

### Patient co-payments

Patient co-payments for healthcare use were estimated based on the 2021 patient fees in Region Västra Götaland, Sweden.^18^ Since co-payments for healthcare are not registered, the estimation had to be made based on template prices and the annual cost ceiling, and with an assumption that the reimbursement period starts at the beginning of the study period. An annual price ceiling of Swedish Crowns (SEK) 1150 is applicable to all publicly provided healthcare services and could thus inform the assumption about maximum annual co-payments for outpatient encounters. Patients incur an additional SEK 100 per night for a hospital stay with a SEK 1500 ceiling over a 30-day consecutive period. An added co-payment of SEK 100 is applied to home visits within primary care. Prescribed pharmaceuticals provided under the benefits scheme are capped at an annual co-payment of SEK 2350^19^ and were obtained for each participant's dispensed medications from the Swedish Prescribed Drug Register.

### Productivity loss

Productivity loss was estimated by multiplying the reported absenteeism from work by the mean wage based on 10-year age categories plus social security contributions.^20 21^ Estimating productivity loss from time spent participating in the intervention was performed using the same method. Baseline wage estimations were calculated using the participant’s age on the date of randomisation.

### Analysis

Demographic characteristics both within and between groups were presented descriptively and comparatively for both the ITT and per-protocol (PP) groups (including participants with at least one PCC phone call with an HCP resulting in a health plan and who used the platform at least once during the intervention). Between-group differences for continuous variables were analysed using the student’s t-test, categorical variables with Pearson’s χ2 test and binary variables with the Fisher’s exact test, using p<0.05 (two-sided) for significance. Individual and household disposable income at randomisation was inflation adjusted to 2021 prices.^22^

Due to the skewness in cost data, average unit costs and total costs were presented as mean values and bootstrapped confidence intervals.^23^ A Students t-test was used to assess statistical differences between the average cost per patient at 2 years. Mean differences in total costs and total effects were used to calculate the incremental cost-effectiveness ratio (ICER). The primary analysis compared the ITT and control group over a 2-year time horizon with both second-year costs and effects discounted at 3% according to recommendations from the Dental and Pharmaceutical Benefits Agency in Sweden.^24^ In addition to societal costs (total costs for drugs, healthcare costs and costs for lost productivity), costs were also reported separately from a patient perspective (co-payments for prescribed drugs and healthcare) and healthcare payer perspective (costs for prescribed drugs and healthcare costs).

Subgroup analyses were performed based on: (1) removing deceased participants and (2) living alone or cohabiting. Sensitivity analyses included: (1) hypothetical UK preference-based value set; (2) second year costs and effects discounted at 5%; (3) second year costs discounted at 3% and effects at 0%; (4) a 1-year time horizon; (5) the control group and the PP group (ie, a minimum of one PCC phone call and used the platform at least once); and (6) removing participants with missing EQ-5D at one of the measurement points. All analyses were performed using R for statistical analysis.^25^

### Public and patient involvement

The PROTECT randomised controlled trial adopted a participatory design, actively involving patients, relatives and HCPs in designing both the study and the digital platform. Extensive community consultations informed the platform’s features, including two-way communication, reliable condition-specific information and user-friendly design, while HCPs advised on layout and technical functionality. An advisory group of patients, relatives, HCPs, developers and researchers collaboratively guided and codesigned all major elements of the study.^11^

## Results

### Participants

The control group consisted of 112 participants and the intervention group of 110 (table 1). There were no statistically significant differences at baseline between the two groups apart from civil status, where a greater percentage was ‘married or living with a partner’ in the control group compared with the intervention group, 77% and 61.8%, respectively. Considerable differences in the mean individual and household disposable income were influenced by the small number of participants.

**Table 1:** Demographic characteristics

Characteristics	Control group (n=112)	Intervention group ITT* (n=110)		Intervention group PP† (n=76)	
	Value	Value	P value	Value	P value
Age (years), mean (SD‡)	70.4 (9.1)	71.1 (9.8)	0.59	70.1 (9.1)	0.84
Women, n (%)	52 (46.4)	51 (46.4)	0.99	31 (40.8)	0.46
**Diagnosis, n (%**)					
CHF§	43 (38.4)	42 (38.2)	0.88	27 (35.5)	0.49
COPD¶	59 (52.7)	56 (50.9)	0.88	38 (50)	0.49
CHD and COPD	10 (8.9)	12 (10.9)	0.88	11 (14.5)	0.49
**Stage of COPD, n (%**)					
Stage 1	16 (27.6)	16 (26.2)	0.99	10 (22.2)	0.94
Stage 2	36 (62.1)	38 (62.3)	0.99	30 (66.7)	0.94
Stage 3	5 (8.6)	6 (9.8)	0.99	4 (8.9)	0.94
Stage 4	1 (1.7)	1 (1.6)	0.99	1 (2.2)	0.94
**Civil status, n (%**)					
Living alone	25 (22.3)	42 (38.2)	0.01	25 (34.2)	0.07
Married or living with partner	87 (77.7)	68 (61.8)	0.01	49 (64.5)	0.07
**Education level, n (%**)					
Compulsory	26 (23.2)	38 (34.5)	0.13	26 (34.2)	0.23
Secondary school	32 (28.6)	25 (22.7)	0.13	18 (23.7)	0.23
Vocational college	21 (18.8)	25 (22.7)	0.13	17 (22.4)	0.23
University	33 (29.5)	22 (20.0)	0.13	15 (19.7)	0.23
**Smoking, n (%**)					
Current smoker	19 (17)	13 (11.8)	0.51	8 (10.5)	0.47
Previous smoker	63 (56.3)	63 (57.3)	0.51	46 (60.5)	0.47
Never smoked	30 (26.8)	34 (30.9)	0.51	22 (28.9)	0.47
**Disposable income, mean (SD**)					
Household** (SEK)	345 560 (243 178)	1 055 249 (6 774 121)	0.27	1 377 027 (8 142 413)	0.27
Individual†† (SEK)‡‡	283 983 (202 230)	1 311 190 (10 200 133)	0.29	1 793 629 (12 265 454)	0.29

* ITT: intention-to-treat.

† PP: per protocol.

‡ SD: standard deviation.

§ CHF: chronic heart failure.

¶ COPD: chronic obstructive pulmonary disease.

** Household: disposable income per unit of consumption of a household, inclusive income earnt in another Nordic country.

†† Individual: disposable income, individual subcomponent, inclusive income earnt in another Nordic country.

‡‡ SEK: Swedish crowns.

### Economic evaluation

**Figure 2:**
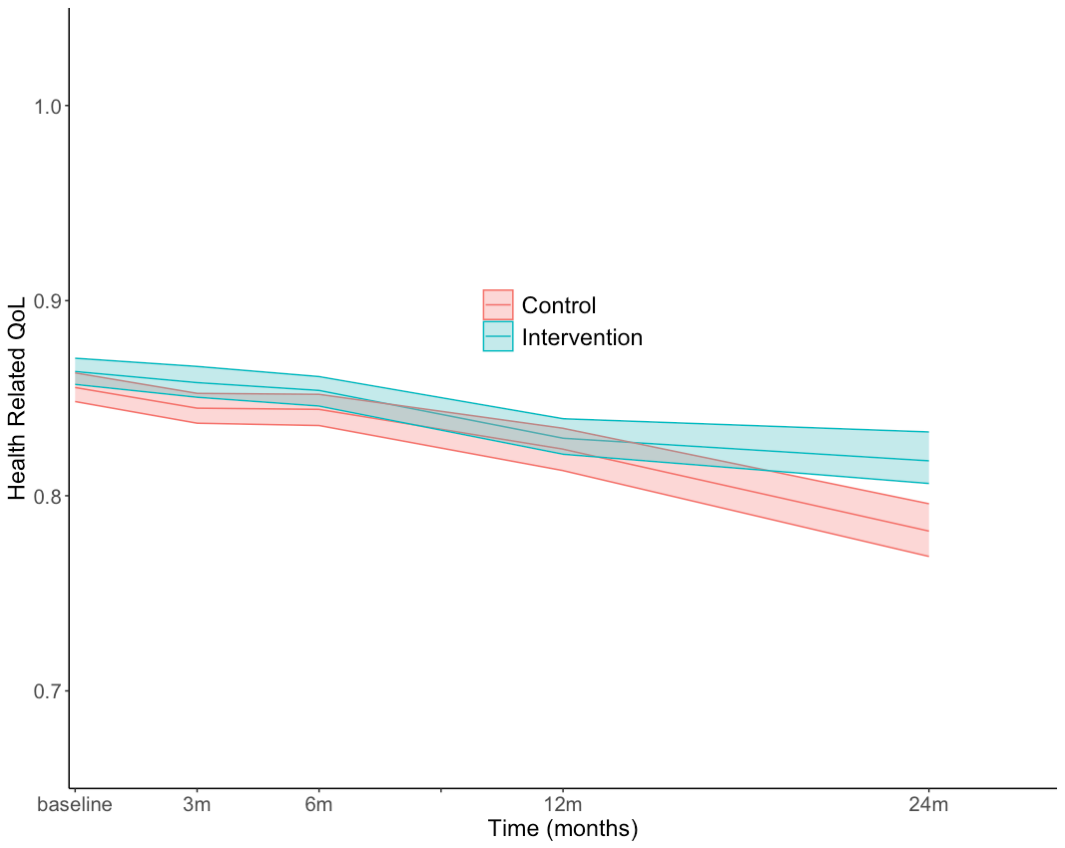
Change in mean imputed health-related quality of life using the Swedish experience-based value set; m, months.

There were no statistically significant differences in self-reported mean imputed HRQoL between groups at baseline, 3, 6, 12 and 24 months after randomisation (online supplemental Table S1). The greatest difference in HRQoL occurred from 12 to 24 months, with the intervention group having an average HRQoL of 0.8179 CI (CI 0.8063 to 0.8327) and the control 0.7819 (CI 0.7689 to 0.7959) (figure 2). The effect difference was 0.0469 QALYs (CI 0.0252 to 0.0686) over 2 years using the Swedish experience-based value set.^14^

At 2 years, the control group had a larger number of specialised outpatient care visits at an average cost per patient of SEK 53 539 (CI 40 386, 64 991) compared with an average of SEK 41 642 (CI 33 173, 49 084) among the intervention patients (table 2 and online supplemental Table S2). Inpatient care costs were SEK 64 899 (CI 40 025, 85 934) per patient in the control group and SEK 44 950 (CI 27 902, 61 111) in the intervention group, driven by the intervention group having fewer overnight hospital admissions (online supplemental Table S3). The intervention group had a higher total cost for polyclinical care compared with the control group as the result of a single surgical procedure not directly related to the study diagnoses, SEK 1 378 612 and SEK 42 234, respectively (online supplemental Table S4). Primary care visits were higher in the intervention group at 4962 compared with 4678 in the control group. The intervention group also spent fewer days on sick or disability pension compared with the control group. The intervention group allocated approximately 317 hours participating in the intervention at a total cost of SEK 176 492 (online supplemental Table S5).

**Table 2:** Average cost per patient at 2 years

Care type	Control (n=112)	Intervention (n=110)	P value
**Primary care, mean (CI**)	35 211 (30 328, 39 776)	36 785 (31 816, 41 578)	0.64
**Inpatient care, mean (CI**)	64 899 (40 025, 85 934)	44 950 (27 902, 61 111)	0.16
**Specialised outpatient care, mean (CI**)	53 539 (40 386, 64 991)	41 642 (33 173, 49 084)	0.12
**Polyclinical care, mean (CI**)	377 (0.0, 1131)	12 533 (897, 34 154)	0.24
**Prescription drugs, mean (CI**)	24 383 (15 695, 30 393)	23 290 (15 582, 28 394)	0.83
**Productivity loss, mean (CI**)	87 139 (26 655, 139 748)	40 776 (404, 70 747)	0.17
**Intervention costs mean (CI**)		1604 (1485, 1722)	–

* Average costs per patient were reported in Swedish Crowns (SEK).

CI, 95% Confidence Interval; HCP, Healthcare Professional; n, number.

**Figure 3:**
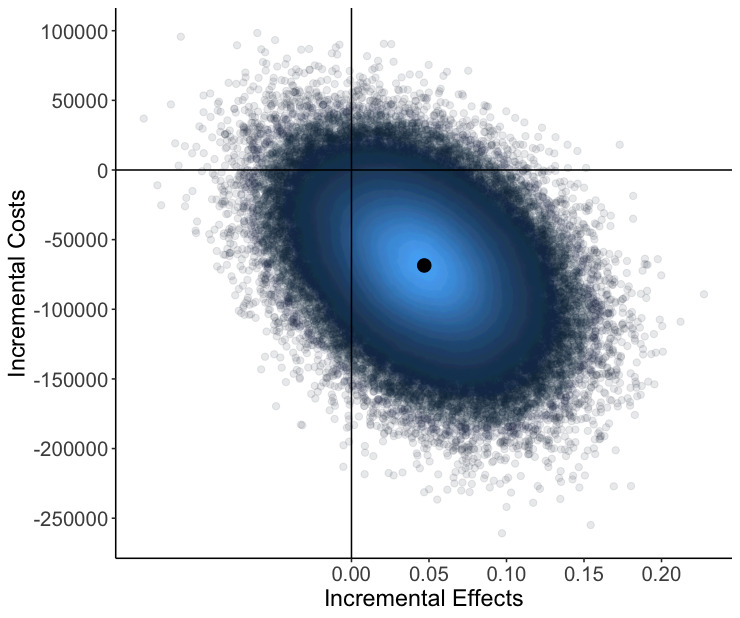
Cost-effectiveness plane, main analysis (bootstrapped). Cost reported in Swedish Crowns (SEK).

The base case cost-effectiveness analysis using the incremental effects of 0.0469 QALYs and the incremental costs of SEK −68 533 (table 3) resulted in the PCC intervention dominating usual care (figure 3).

**Table 3:** Cost-effectiveness analysis

Analysis type	Effect difference (mean, CI)	Cost difference (mean/CI)	ICER
**Swedish value set 3% discounting**	0.0469 (0.0252, 0.0686)	−68 533.12 (−86 827.63, to 50 238.6)	Dominant

CI, Confidence Interval; ICER, incremental cost-effectiveness ratio.

### Sensitivity analysis

Sensitivity analysis using the hypothetical UK preference-based value set resulted in an incremental effect of 0.0961 QALYs and an incremental cost difference of SEK −68 372 (online supplemental Table S6) Remaining sensitivity analyses were conducted with the Swedish experience-based value set. Comparison of the PP group compared with the control group yielded an incremental effect of 0.0793 QALYs and a cost difference of SEK −79 578. Analysis after 12 months showed an incremental effect size of 0.0166 QALYs and incremental costs SEK −47 832. Discounting second year costs at 3% and effects at 0% and both costs and effects at 5% showed no difference compared with the primary analysis. Removing participants with missing EQ-5D results at 3, 6, 12 and 24 months resulted in an incremental effect of 0.0119 QALYs and incremental costs of SEK −42 156.

### Subgroup analysis

Subgroup analysis of participants who were ‘married or living with a partner’ resulted in an incremental effect of 0.0242 QALYs and incremental costs of SEK −39 877 (online supplemental Table S7). Comparison of participants who were ‘living alone’ had an incremental effect of 0.0640 QALYs and the incremental costs of SEK −164 071. Removing deceased patients from the analysis lowered incremental effects to 0.0378 QALYs with incremental costs of SEK −85 657.

### Healthcare and patient perspective

The healthcare payer perspective resulted in total costs of SEK 18 803 977 for the control group and SEK 16 986 442 for the intervention group, following the same trajectory as the societal perspective analysis accounting only for direct healthcare and prescription drug costs (online supplemental Table S8). Based on patient co-payments (online supplemental Table S9), average healthcare costs between year 1 and 2 decreased from SEK 896 to SEK 802 per person in the intervention group and from SEK 940 to SEK 863 in the control group. Forty-two patients in the intervention group had a total of 268 hospital admission nights in year 1, reducing to 35 patients and a total of 243 nights in year 2. The control group had 56 patients for a total of 318 nights in year 1, also reducing in the second year to 43 patients and 322 overnight admissions. The time spent in the intervention corresponded to hypothetical productivity losses of SEK 1367 per intervention participant (online supplemental Table S10).

## Discussion

The results of the study showed that participants with CHF and/or COPD who had access to the remote PCC add-on intervention had a slower decrease in HRQoL, less healthcare utilisation and lower costs compared with those provided usual care over a 2-year time horizon. The intervention group had less utilisation of specialised outpatient care and inpatient care, fewer overnight hospital admissions and had less productivity loss.

The application of PCC in the provision of care for patients with chronic conditions has shown positive effects in previous economic evaluations.^26^,^29^ Examples of this are patients diagnosed with acute coronary syndrome, where PCC was cost-effective for patients under 65,^27^ within palliative homecare^29^ and in patients with hip fractures.^28^ There are, however, relatively few studies that have evaluated the economic benefits of PCC administered through different eHealth mediums. One previous study^26^ that combined PCC with telehealth for hospitalised patients with COPD and/or CHF reported incremental effects of 0.018 QALYs and incremental costs of SEK −12 434 at 6 months. The results of this study further build the knowledge of cost-effectiveness research within PCC through the added application of a digital tool with patients in a primary care setting. By addressing the needs of patients living with chronic conditions within a primary care setting, demand on other services such as inpatient and outpatient care may be reduced. This not only highlights the benefit for chronic conditions but also demonstrates the wider benefits PCC can have when applied across the entire healthcare system, regardless of disease.

Health economic evaluations adopt different perspectives when determining the types of health benefits and costs to be included. At its most basic, a societal perspective includes all appropriate healthcare costs, as well as costs associated with productivity loss that arise from a patient’s inability to work.^30^ A societal perspective was applied to this economic evaluation estimating total costs for productivity loss of SEK 4 485 378 in the intervention group and SEK 9 759 578 in the control group. A criticism of this approach is that it often fails to account for the effects of productivity loss in older patients who no longer participate in the labour market, such as unpaid work, including informal caregiving, household production activities and volunteer work.^31^ Costs related to lost productivity account for approximately 40% of the total healthcare costs for patients with COPD and/or CHF that still participate in the labour market; however, information on the long-term effects after retirement is limited.^5 32^ Within this study, stratified sampling, using an estimated retirement age of 65 years, allocated n=89 participants to each of the intervention and the control group. Within this total population of n=178 participants, eight still participated in the labour market, with only two participants working more than 75%. With such a large number of participants having no calculated productivity loss, there is a risk of under-valuing the true cost-effectiveness of interventions for older patients living with chronic conditions.

Sensitivity analysis using the hypothetical UK preference-based value set (UK value set) showed an increase in incremental effects of 0.0961 QALYs compared with the Swedish experienced-based value set, 0.0469 QALYs. The UK value set is developed based on hypothetical evaluations from the public and is recommended by the National Institute of Health and Clinical Excellence as a more representative value within collectively financed healthcare systems.^33^ The Swedish experience-based value set, however, comes from individuals who have evaluated their own health, and as Dolan argues,^34^ it more accurately represents the real suffering of patients. However, Neumann *et al*^35^ highlights that a patient’s ability to develop coping mechanisms and accommodate their limitations may underestimate the true health value associated with their disease, a limitation of experience-based measures. Within PCC, the patients’ experiences are central and the basis for discussions with HCPs alongside objective markers; therefore, one could argue for the relevance of experience-based value sets. As tools for demonstrating the robustness of results when informing decision-makers, the reporting of both value sets should be considered as well as their relevance to the population, condition and intervention type being investigated.

### Strengths and limitations

Among the strengths of the study are the use of a randomised controlled trial design that reduces the risk of bias,^36^ and the use of near complete registers at both the national and regional level that provided a detailed overview of the costs and effects of illness. Furthermore, reporting of costs from both a societal perspective in the cost-effectiveness analysis and descriptively from the patient perspective is in line with the values of PCC research and considered a strength to this study.

Limitations within the study were related to missing EQ-5D values and the accuracy of cost estimations. first, both the intervention and control group had missing EQ-5D data at each of the measurement points; however, the intervention group had almost twice as many missing values at 3, 6, 12 and 24 months. A qualitative analysis^37^ of the original RCT that investigated meaningful use of the platform revealed that participants generally felt they already had their health under control and had access to the necessary support. The research team believes this may have accounted for the greater number of missing EQ-5D data in the intervention group. Advanced imputation methods and bootstrapping were applied to the data to address uncertainty in the final effect and cost estimates as well as a sensitivity analysis showing reduced incremental HRQoL and incremental costs when participants with missing EQ-5D were removed. Additionally, it should be noted that even though the HRQoL scores decreased more slowly in the intervention group, only at 24 months can the difference between groups be regarded as reaching a minimal important clinical difference, HRQoL difference of 0.0357. However, this is based on minimal important clinical difference estimations using the UK value set, a limitation of the Swedish experience-based value set.^38^ Second, cost data for primary care were collected from regional guidelines that estimate costs based on weighted contacts,^15^ which may not accurately reflect healthcare costs and utilisation, and do not clearly account for patient co-payments. Additionally, of 3036 registered visits to specialised outpatient care, 712 DRG weights were missing, which were estimated using the same method as the primary care. Finally, the imputation of missing time within the intervention logbooks corresponds to approximately 130 min over the entire study population, which would have little to no effect on the final cost calculation. To account for possible limitations with the data, multiple sensitivity and subgroup analyses were performed that showed little deviation from the results of the primary analysis.

## Conclusions

The results demonstrated that a remote PCC add-on intervention for people with COPD and/or CHF was cost-effective and dominated usual care, with the intervention group having both lower healthcare costs and higher HRQoL than the participants in the control group. Adopting remote digital PCC programmes could prove valuable in combating rising healthcare costs of chronic illnesses such as COPD and CHF that are placing an increased strain on healthcare systems globally.

## Supplementary material

10.1136/bmjopen-2024-093083 online supplemental file 1

## Data Availability

Data may be obtained from a third party and are not publicly available.
