# Supplementary material for: Economic evaluation of a person-centred care intervention with a digital platform and structured telephone support for people with chronic heart failure and/or chronic obstructive pulmonary disease: results from a randomised controlled trial in Sweden
Source: BMJ Open. 2025 Oct 9;15(10):e093083. doi: 10.1136/bmjopen-2024-093083 (PMC12516990; doi:10.1136/bmjopen-2024-093083)
Supplement: online supplemental file 1 [file bmjopen-15-10-s001.docx]

**Economic evaluation of a person-centred care intervention with a digital platform and structured telephone support for people with chronic heart failure and/or chronic obstructive pulmonary disease: results from a randomised controlled trial in Sweden**

**Author names and affiliations:**

Authors: Harvey B P.^1,2^, Barenfeld E^1,2,3^, Fors A^1,2,4^, Ekman I^1,2,5^, Swedberg K^2,6^, Gyllensten H^1,2^

**Institution where the work was performed:**

Institute of Health and Care Sciences, University of Gothenburg, Sweden.

**Affiliations**

^1^ Institute of Health and Care Sciences, University of Gothenburg, Box 457, SE-405 30 Gothenburg, Sweden.

^2^ University of Gothenburg Centre for Person-Centred Care (GPCC), Sahlgrenska Academy, University of Gothenburg, Sweden.

^3^ Institute of Neuroscience and Physiology, Department of Health and Rehabilitation, Sahlgrenska Academy, University of Gothenburg, Box 455, SE-40530 Gothenburg, Sweden.

^4^ Region Västra Götaland, Research, Education, Development and Innovation, Primary Health Care, Gothenburg, Sweden.

^5^ Department of Medicine, Geriatrics and Emergency Medicine, Sahlgrenska University Hospital/Östra, Gothenburg, Sweden

^6^ Department of Molecular and Clinical Medicine, Sahlgrenska Academy, Gothenburg, Sweden.

Content

[*Supplementary Material 1.* 4](#_Toc205993887)

[Table S1. Mean imputed Health Related Quality of Life 5](#_Toc205993888)

[Table S2. Unit Costs 6](#_Toc205993889)

[Table S5. Healthcare Professionals Intervention Costs (6 months) 11](#_Toc205993890)

[Table S6. Sensitivity analyses 12](#_Toc205993891)

[Table S7. Subgroup Analysis 13](#_Toc205993892)

[Table S8. Healthcare Payer Perspective Year Two 14](#_Toc205993893)

[Table S9. Patient Co-payment Prices (6) 15](#_Toc205993894)

[Table S10. Intervention Costs (Participants) (4,5) 16](#_Toc205993895)

[References 17](#_Toc205993896)

***Supplementary Material 1.***

***Multiple Imputation***

At any measurement point, a single missing response to the EQ-5D questionnaire would result in the inability to calculate the utility weights. A commonly proposed method for dealing with ‘missingness’ related to observed data is to apply multiple imputation. Multiple imputation addresses this issue by creating multiple datasets with plausible data, allowing researchers to explicitly incorporate the uncertainty about the true value (22). Missing EQ-5D values at baseline were imputed using mean imputation independent of treatment allocation. Mean imputation has been recommended as more efficient than multiple imputation at baseline as it ensures that impute values are equally distributed amongst the population and independent of treatment allocation (23). After stratifying by treatment group, 100 imputed data sets were generated using predictive mean matching with the Multivariate Imputation by Chained Equations R package (24). The imputed data sets were combined and the sum of the total costs for year one and two were calculated. Linear interpolation method was used to calculate QALYs, meaning that the utility value for the consecutive time points is multiplied by the time between the two time points expressed in years, after which the results of all periods are summed to calculate total QALYs. Both total costs and total QALYs were regressed using seemingly unrelated regression based on population, diagnosis, sex, age, education level, previous angina, previous heart attack, smoker, previous diabetes, previous stroke, previous hypertension, and civil status. Seeming unrelated regression is a multivariate regression model that is fitted on each bootstrap sample to return the estimated adjusted regression coefficients of the independent variable (25). This is done for each of the 100 imputed datasets where non-parametric bootstrapping was set to result in 1000 bootstrap samples per imputed dataset. Rubin’s rule is applied to pool the results of the imputed datasets that are calculated in the incremental cost-effectiveness ratio (ICER).

# Table S1. Mean imputed Health Related Quality of Life

| **Measurement Point** | **Intervention (mean, CI)** | **Control  (mean, CI)** | ***P* value** |
| --- | --- | --- | --- |
| **Baseline** | 0.8637  (0.8571, 0.8705) | 0.8555  (0.8483, 0.8630) | 0.72 |
| **3 months** | 0.8580  (0.8505, 0.8663) | 0.8449  (0.8372, 0.8525) | 0.45 |
| **6 months** | 0.8540  (0.8460, 0.8611) | 0.8443  (0.8360, 0.8520) | 0.93 |
| **12 months** | 0.8295 ( 0.8213, 0.8395) | 0.8239  (0.8129, 0.8346) | 0.77 |
| **24 months** | 0.8179  (0.8063, 0.8327) | 0.7819  (0.7689, 0.7959) | 0.34 |

CI: 95% Confidence Interval

# Table S2. Unit Costs

| **Type of visit** | **Unit cost ^1^** | **Year 1** | | **Year 2** | |
| --- | --- | --- | --- | --- | --- |
|  |  | Control | Intervention | Control | Intervention |
|  |  | **Total Units** | | | |
| **Primary Care (1,2)** |  |  |  |  |  |
| ***Direct contact:*** |  |  |  |  |  |
| *Physician* | 2 265 | 330 | 312 | 288 | 275 |
| *Physician - home visit* | 4 530 | 6 | 2 | 10 | 1 |
| *Other (nurse, physio etc.)* | 906 | 759 | 880 | 601 | 670 |
| *Other - team visit* | 1 359 | 3 | 1 | 1 | 0 |
| *Other - group visit* | 906 | 10 | 13 | 14 | 20 |
| *Other - group/team visit* | 1 359 | 6 | 0 | 0 | 0 |
| ***Indirect contact:*** |  |  |  |  |  |
| *Physician* | 755 | 459 | 429 | 409 | 420 |
| *Physician - home visit* | 1 510 | 4 | 19 | 4 | 15 |
| *Other (nurse, physio etc.)* | 302 | 764 | 880 | 1005 | 1024 |
| *Other - team visit* | 453 | 3 | 0 | 1 | 0 |
| *Other - group visit* | 302 | 0 | 0 | 0 | 1 |
| *Other - combined group & team visit* | 453 | 1 | 0 | 0 | 0 |
| **Total Units:** | | **2345** | **2536** | **2333** | **2426** |
| **Inpatient Care (3)** | | | | | |
| *Inpatient care costs (mean, CI)* | 69 393  (62 538, 76 848) | **56** | **43** | **42** | **35** |
| **Specialised Outpatient Care (1,3)** | | | | | |
| *Specialised care cost DRG (mean, CI)* | 3852  (3754, 3971) | 664 | 503 | 675 | 482 |
| ***Direct contact*** |  |  |  |  |  |
| *Physician* | 4 515 | 50 | 54 | 50 | 51 |
| *Physician - team visit* | 6 772 | 0 | 1 | 2 | 1 |
| *Other (nurse, dietitian etc.)* | 1 806 | 35 | 35 | 23 | 52 |
| *Other - team visit* | 2 709 | 2 | 8 | 5 | 0 |
| *Other - combined group visit* | 1 806 | 0 | 7 | 0 | 1 |
| ***Indirect contact*** |  |  |  |  |  |
| *Physician* | 1 505 | 34 | 28 | 71 | 39 |
| *Other (nurse, dietitian etc.)* | 602 | 29 | 38 | 40 | 56 |
| **Total:** | | **814** | **674** | **866** | **682** |
| **Polyclinical Care (3)** | | | | | |
| *Polyclinical care cost (mean, CI)* | 236 808  (43 076, 595 715) | **1** | **2** | **0** | **3** |
| **Prescription Drugs** |  |  |  |  |  |
| *Total Pharmaceutical costs (mean, CI)* | 286  (260.4, 316.2) | **4486** | **4568** | **4296** | **5178** |
| **Absenteeism^2^ (4,5)** | | | | | |
| *25 - 34 years of age* | 1 374 | 0 | 365 | 0 | 365 |
| *35 - 44 years of age* | 1 612 | 0 | 365 | 0 | 365 |
| *45 - 54 years of age* | 1 728 | 1204 | 178 | 1113 | 247 |
| *44 - 64 years of age* | 1 672 | 2196 | 730 | 2076 | 835 |
| **Total:** | | **3400** | **1638** | **3189** | **1812** |

^1^ All unit costs were reported in Swedish Crowns (SEK), ^2^ CI: 95% Confidence Interval, ^3^ Sick leave units are average cost per day.

Table S3. Patient co-payment for inpatient and outpatient care / Patient fees for hospital nights

|  | **Year 1** | | **Year 2** | |
| --- | --- | --- | --- | --- |
|  | Control | Intervention | Control | Intervention |
| **Patient Co-Payment (Primary, Specialised Outpatient, Polyclinical, Inpatient) (6)** | | | | |
| Patient Co-payment (mean, CI) | 940  (881.5, 999.5) | 896  (834.1, 962.2) | 863 (798.6, 929.7) | 802  (728.2, 879.0) |
| Total Costs (SEK)^1^ | 104 350 | 98 550 | 95 750 | 88 250 |
| **Patient Co-Payment (Inpatient Care) (6)** | | | | |
| Patients (n = number) | n=56 | n=42 | n=43 | n=35 |
| Hospital nights (n = nights) | n=318 | n=268 | n=322 | n=243 |
| Patient Hospital Co-payment (mean, CI) | 508.9  (359.0, 639.2) | 571.4  (431.0, 702.3) | 548.5  (472.1, 807.0) | 548.6  (368.6, 711.4) |
| Total Costs (SEK)^2^ | 28 500 | 24 000 | 27 900 | 19 200 |
| **Patient Co-Payment (Prescription Medication) (7)** | | | | |
| Patient Co-payment (mean, CI) | 6197* ( 2145, 17847) | 2164 (1970, 2341) | 2268 (2100, 2434) | 2145 (1959, 2315) |
| Total Costs (SEK)^3^ | 681 615 | 238 021 | 247 174 | 233 824 |

^1^ Total costs reported based on a co-payment ceiling of SEK 1150, ^2^ Total cost reported based on a payment ceiling of SEK 1500 over a consecutive 30-day period, ^3^ Total cost reported based on a price ceiling of SEK 2350 for prescribed medications provided in the benefit, CI: 95% Confidence Interval, SEK: Swedish Crowns, n: number, *mean for the control group in year one is effected by 3 payments of medications outside of the financial benefits scheme.

Table S4. Total Costs at year 1 and year 2

| **Type of visit** | **Year 1** | | **Year 2** | |
| --- | --- | --- | --- | --- |
|  | **Control** | **Intervention** | **Control** | **Intervention** |
| **Primary Care** | 2 066 888 | 2 141 784 | 3 943 667 | 4 046 347 |
| **Inpatient Care** | 4 116 808 | 2 801 098 | 7 268 665 | 4 944 473 |
| **Specialised Outpatient Care** | 2 858 651 | 2 282 809 | 5 996 345 | 4 580 664 |
| **Polyclinical Care** | 42 234 | 153 720 | 42 234 | 1 378 612 |
| **Prescription Drugs** | 1 486 028 | 1 262 627 | 2 730 855 | 2 561 899 |
| **Productivity Loss** | 5 154 856 | 2 160 214 | 9 759 578 | 4 485 378 |
| **Intervention Costs** |  | 176 291 |  | 176 292 |

* Total costs were reported in Swedish Crowns (SEK)

# Table S5. Healthcare Professionals Intervention Costs (6 months)

| **Type of visit** | **Unit cost** | **HCP Intervention Costs** | | | | |
| --- | --- | --- | --- | --- | --- | --- |
|  | **SEK/per hour** | **Number of Occasions** | **Average time (min)** | **Imputed time over intervention period (min)** | **Total time (hrs)** | **Total Cost**  **(SEK)** |
| Health-plan calls (initial) | 557 | 108 | 31 | 3300 | 55 | 30 635 |
| Health-plan calls (follow-up) | 557 | 255 | 20 | 5021 | 84 | 46 788 |
| Introduction and digital support | 557 | 110^1^ | 13.5^2^ | 1485 | 25 | 13 786 |
| Health care support | 557 | 20 + 12 imp^3^ | 9 | 288 | 5 | 2 785 |
| Formulating and documenting health plans | 557 | 317 | 17 | 5405 | 90 | 50 130 |
| Sending messages and emails | 557 | 386 | 3 | 1158 | 19 | 10 583 |
| Maintenance Costs | 557 | 26 | 90 | 2340 | 39 | 21 723 |
|  |  |  |  |  | Total: | 176 429 |

^1^ Number of individuals, ^2^ Per-person average time (corresponds to 1.4 contacts per person), ^3^ 12 imputed health care support conversations due to missing data, min = minutes, hrs =hours, SEK = Swedish Crowns

# Table S6. Sensitivity analyses

| **Analysis Type** | **Effect Difference (mean, CI)** | **Cost Difference (mean/CI)** | **ICER** |
| --- | --- | --- | --- |
| **UK value set 3% discounting** | 0.0961  (0.0594, 0.1328) | -68372.09  (-86645.21, -50098.97) | Dominant |
| **Per Protocol 3% discounting** | 0.0793 (0.0572, 0.1014) | -79587.56 (-100570.2, -58604.89) | Dominant |
| **Swedish Value set Year 1** | 0.0166 (0.0081, 0.0251) | -47832.21 (-58909.57, -36754.86) | Dominant |
| **Swedish value set (0% & 3% discounting)** | 0.0479 (0.0257, 0.0700) | -68533.12  (-86827.63, -50238.6) | Dominant |
| **Swedish value set (5% discounting)** | 0.0463 (0.0249, 0.0677) | -68073.06 (-86181.41, -49964.71) | Dominant |
| **Missing EQ-5D Removed** | 0.0119 (0.0259, -0.0021) | -42156.13 (-60401.74, -23910.52) | Dominant |

CI: 95% Confidence Interval, ICER: Incremental Cost-Effectiveness Ratio, UK: United Kingdom

| **Analysis Type** | **Effect Difference (mean, CI)** | **Cost Differenced (mean/CI)** | **ICER** |
| --- | --- | --- | --- |
| **Swedish value set 3% discounting  (Deceased Removed)** | 0.0378 (0.0219, 0.0537) | -85656.76  (-103111.9, -68201.63) | Dominant |
| **Cohabiting 3%** | 0.0242  (0.002, 0.0467) | -39876.94 (-59244.36, -20509.52) | Dominant |
| **Living Alone 3% discounting** | 0.0640 (0.0224, 0.1056) | -164070.5  (-212612.4, -115528.6) | Dominant |

# Table S7. Subgroup Analysis

CI: 95% Confidence Interval, ICER: Incremental Cost-Effectiveness Ratio, UK: United Kingdom

| **Type of visit** | **Year 2** | |
| --- | --- | --- |
|  | **Control** | **Intervention** |
| Primary Care | 3 943 667 | 4 046 347 |
| Inpatient Care | 7 268 665 | 4 944 473 |
| Specialised Outpatient Care | 5 996 345 | 4 580 664 |
| Polyclinical Care | 42 234 | 1 378 612 |
| Prescription Drugs | 2 730 855 | 2 561 899 |
| Intervention Costs |  | 176 292 |
| **Total Direct Healthcare Costs:** | 19 981 766 | 17 688 287 |
| **Minus** | | |
| **Total Co-payments:** | 1 177 789 | 701 845 |
|  | | |
| **Healthcare Payer Perspective Cost:** | 18 803 977 | 16 986 442 |

# Table S8. Healthcare Payer Perspective Year Two

* Total costs were reported in Swedish Crowns (SEK)

# Table S9. Patient Co-payment Prices (6)

|  | Fee (SEK) | Description |
| --- | --- | --- |
| **Primary Care** | 100 | Physician visit at registered healthcare centre |
|  | 50 | Visit to another registered healthcare professional (e.g. nurse, physiotherapist, occupational therapist, team, and group visits) |
|  | 100 | Additional fee for home visits |
| **Inpatient** | 300 | Hospital visit co-payment |
|  | 100 | Price per hospital night (maximum of SEK1500 for each 30-day period) |
| **Specialised Outpatient Care** | 300 | Visit to a specialist physician |
| **Poly-Clinical Care** | 300 | Visit to a specialist physician |

SEK: Swedish Crowns

# Table S10. Intervention Costs (Participants) (4,5)

| **Type of visit** | **Unit cost** | **Intervention costs participants** | | |
| --- | --- | --- | --- | --- |
| **Age group categories** | **SEK (hrs)** | **Total time (min)** | **Total time (hrs)** | **Total cost (SEK)** |
| *25 - 34 years of age (n=1)* | 241 | 295.05 | 4.92 | 1 186 |
| *45 - 54 years of age (n=6)* | 303 | 813.15 | 13.56 | 4 109 |
| *44 - 64 years of age (n=13)* | 293 | 5 230.55 | 87.18 | 25 544 |
| *65 + years of age (n=89)* | 238 | 29 776.6 | 496.3 | 118 119 |
| **Total (n=109*)** |  | 36 115.4 | 602.0 | 148 958 |
| **Mean cost per participant** |  |  |  | 1367 |
|  |  |  |  |  |

min = minutes, hrs =hours, SEK = Swedish Crowns

# **References**

1. Swedish Association of Local Authorities and Regions. Statistik om hälso och sjukvård samt regional utveckling 2021 [Statistics on healthcare and regional development 2021]. [cited 2024 Mar 4]; Available from: https://skr.se/skr/halsasjukvard/ekonomiavgifter/ekonomiochverksamhetsstatistik.46542.html

2. Region Västra Götland. Teambesök [Team visit]. 2017 Apr 10 [cited 2024 Feb 17]; Available from: https://www.vgregion.se/halsa-och-vard/vardgivarwebben/vardadministration/patientavgiftshandboken/oppenvard/teambesok/

3. National Board for Health and Welfare. Viktlistor för NordDRG [Weight list for NordDRG]. 2022 Nov 9 [cited 2023 Jun 14]; Available from: https://www.socialstyrelsen.se/statistik-och-data/klassifikationer-och-koder/drg/viktlistor/

4. Statistics Sweden. Genomsnittlig grundlön, kronor efter sektor, yrkesgrupper (SSYK 2012), kön, ålder och år [Statistical database: Average wage, SEK by sector, occupational group (SSYK 2012), sex, age, and year). In: Statistical Database [Internet]. [cited 2024 Feb 23]. Available from: http://www.statistikdatabasen.scb.se/pxweb/sv/ssd/START__AM__AM0110__AM0110A/LonYrkeAlder4A/

5. The Swedish Tax Agency. Employer Contributions. [cited 2024 Feb 23]; Available from: https://skatteverket.se/servicelankar/otherlanguages/inenglishengelska/businessesandemployers/startingandrunningaswedishbusiness/declaringtaxesbusinesses/filingapayetaxreturn/employercontributions.4.2fb39afe18dabf1e4d24a3d.html

6. Swedish Association of Local Authorities and Regions. Patientavgifter i öppen hälso- och sjukvård år 2021 [Patient fees in primary care year 2021]. [cited 2023 Nov 11]; Available from: https://skr.se/download/18.71b542201784abfbf7a124e9/1631690554988/avgift_oppen_slutenvard_2021_uppdaterad.pdf

7. Swedish eHealth Agency. Högkostnadsskydd Läkemedel [National Ceiling Prescribed Drugs]. [cited 2024 Mar 3]; Available from: https://www.ehalsomyndigheten.se/privat/e-recept/hogkostnadsskydd/
