# Supplementary material for: Economic evaluation of a person-centred care intervention with a digital platform and structured telephone support for people with chronic heart failure and/or chronic obstructive pulmonary disease: results from a randomised controlled trial in Sweden
Source: BMJ Open. 2025 Oct 9;15(10):e093083. doi: 10.1136/bmjopen-2024-093083 (PMC12516990; doi:10.1136/bmjopen-2024-093083)
Supplement: Supplementary file 2 [file bmjopen-2024-093083.reviewer_comments.pdf]

PEER REVIEW HISTORY

BMJ Open publishes all reviews undertaken for accepted manuscripts. Reviewers are asked to complete a checklist review form and are provided with free text boxes to elaborate on their assessment. These free text comments are reproduced below.

ARTICLE DETAILS

Title (Provisional)

Economic evaluation of a person-centred care intervention with a digital platform and structured telephone support for people with chronic heart failure and/or chronic obstructive pulmonary disease: results from a randomised controlled trial in Sweden

Authors

Harvey, Benjamin Peter; Barenfeld, Emmelie; Fors, Andreas; ekman, inger; Swedberg, Karl; Gyllensten, Hanna

---

VERSION 1 - REVIEW

Reviewer 1

Name Magalhaes, Teresa

Affiliation NOVA University Lisbon, NOVA National School of Public Health

Date 24-Jan-2025

COI None

The paper brings a knowledge that is scarce in this area.

Congratulations for your work. The design of the investigation is very well done as well the paper writing.

There is just one detail in the methodology on the healthcare costs that should be better explained. On page 9 between line 20 and 26 the different options need to be better justified.

Reviewer 2

Name Jarl, Johan

Affiliation Lund University, Health Economics Unit, Department of Clinical Science (Malmö)

Date 28-Apr-2025

COI None

The authors have conducted an economic evaluation of a remote person-centred care add-on intervention for persons with chronic heart failure and/or chronic constructive pulmonary disease. The data comes from a randomised controlled trial. The evaluation is competently done and presented in a clear manner. However, I have some questions/comments.

Concerns

• Participants – please add in text how many of the assessed were eligible as this is the number of interest with regard to participation.

• The impact of productivity losses with regard to retirement might impact the results unless the distribution of the two groups are perfectly balanced with regard to age/retirement. It's currently not possible to see this from the presented data (on page 15 it is reported that 89 participants across both groups are above 65 years of age). One way to deal with this would be to include an additional subgroup analysis based on being working aged/retired.

• I am confused about the inclusion of patient fees. They should not be included in the societal perspective as the full resource utilisation is included in the healthcare costs. However, patient fees are not included in the cost table, so it seems that it is, correctly, excluded. But then, what is the purpose of the section on patient fees in the method and the last paragraph in the result section? In the "Strengths and limitations", the authors allude to some form of "patient perspective", but this is unclear how this has been analysed. If it is a part of the purpose of the study, it would help the reader if this was clarified, and a part of the aim.

• The difference in effect is not clearly stated. In the result section, it is presented as HRQoL (0.0469). This is later reported as a QALY difference. Please clarify if this number is the difference in accumulated QALY over the two years or if it is a difference in health state utility. This would potentially be clearer if Table S1 also showed the calculated QALY.

• Clarify if the costs are significantly different between the groups, or the different cost categories.

Minor

• Title: "Economic evaluation of person-centred care using a digital platform…" is somewhat misleading. It is not an evaluation of person-centred care, but a specific support system that aims to achieve person-centred care.

• A "registry" is where registers are kept. That is, "registry" and "registers" are not interchangeable words.

• Add the perspective to your aim

• Introduction (p5, line 13): "innovate current methods" – current methods need not be innovated. I guess you mean to "innovate on" or something along those lines.

• P8 line 6: typo "listed created"

• “Healthcare costs” explain how the authors have obtained primary care and specialised outpatient care costs when DRG weights are missing. But it does not say how they moved from DRG to costs when it was available. I assume in a manner similar to inpatient costs but would be good if this was clarified.

• P 14 line 21: typo “and”

• Page 15 line 27: Here “indirect costs” are introduced for the first time. It is unclear what this refers to. Productivity losses?

• Table 2: The intervention cost is presumably the SEK176’492 as presented in the text. But 176492/110=1604.

Suggestion

• Since you have access to healthcare costs, it would be interesting if you also presented the results from the healthcare perspective (payer), as this would be important information for a decision maker and make the results accessible also in contexts where productivity losses are considered inappropriate in economic evaluations. It would also deal with some of the problems you discuss regarding including productivity losses. I do think you are correct in including productivity losses but excluding them would show how this choice influences the conclusion.

VERSION 1 - AUTHOR RESPONSE

Reviewer 1:

The paper brings a knowledge that is scarce in this area. Congratulations for your work. The design of the investigation is very well done as well the paper writing.

There is just one detail in the methodology on the healthcare costs that should be better explained. On page 9 between line 20 and 26 the different options need to be better justified.	In order to more adequately justify the “Healthcare Costs” paragraph we have emphasised that for Primary Care, for which the cost calculations are described between line 20-26, it is recommended to use the price estimations provided by the Swedish Association of Local Authorities and Region’s Statistics for Healthcare and Regional Development 2021. Below is the original paragraph, followed by the amended paragraph that describes the process more clearly.<br><br>**Page 9, Lines 24-26 & Page 10, Lines 1-16**<br><br>**Previous Paragraph:**<br><br>**Healthcare Costs**

	Unit costs for primary care and specialised outpatient care, when the DRGs weights were missing, were calculated using average unit price estimations from the Swedish Association of Local Authorities and Region's Statistics for Healthcare and Regional Development 2021 (15). Costs for primary care were calculated using the unit cost for a consultation with a physician. Other HCPs (registered nurse, occupational therapist, physiotherapist etc.) and group visits were priced at 40% the cost of a physician. Team and Group/Team visits, where a patient meets a 'care team', were defined as utilising more than one HCP and priced at one and a half times the cost of a single HCP (Physician or other HCP) (16). Care provided in the home was two times the unit cost of a normal consultation to an HCP. Indirect care (telephone consultation) was priced at one-third of the unit cost of the performing HCP. Per-patient total healthcare costs for inpatient care, polyclinical care and specialised outpatient care were calculated using the 2017-2021 DRG weights, times the average cost per healthcare visit in 2021 (17). Missing DRG weights for specialised outpatient care were calculated using the same method applied to primary care, however, using the average costs of a specialist consultation in 2021 (15). Healthcare utilisation was calculated using 2021 prices and therefore not inflation adjusted. Amounts and costs of dispensed pharmaceuticals were calculated using the total cost excluding taxes. The direct costs for HCPs salaries were calculated using the mean hourly wage and social contributions (personal communication, Glenn Sandvik 2023-06-07), multiplied by the total hours spent in patient-facing meetings, documentation, and communication as part of the intervention.<br><br>**New Paragraph:**<br><br>**Healthcare Costs**<br><br>Unit costs for primary care were calculated using the average unit price estimations recommended by the Swedish Association of Local Authorities and Region's Statistics for Healthcare and Regional Development, 2021 (15). All costs were calculated with respect to the performing HCP (Physician or

	Other HCP i.e. registered nurse, occupational therapist, physiotherapist etc.). Other HCP and group visits were priced at 40% the cost of a consultation with a physician. Team and Group/Team visits, where a patient meets a ‘care team’, were defined as utilising more than one HCP and priced at one and a half times the cost of a consultation (16). Care provided in the home was calculated at two times the unit cost of a consultation. Indirect care (telephone consultation) was priced at one-third of the unit cost of a consultation.<br><br>Per-patient total healthcare costs for inpatient care, polyclinical care and specialised outpatient care were calculated using the 2017-2021 DRG weights, times the average cost per healthcare visit in 2021 (17). Missing DRG weights for specialised outpatient care were calculated using the same method applied to primary care, however, using the average cost of a specialist consultation in 2021 (15). Polyclinical care and inpatient care had complete DRG information and therefore no additional imputations were required. Healthcare utilisation was calculated using 2021 prices and therefore not inflation adjusted. Amounts and costs of dispensed pharmaceuticals were calculated using the total cost excluding taxes. The direct costs for HCPs salaries were calculated using the mean hourly wage and social contributions (personal communication, Glenn Sandvik 2023-06-07), multiplied by the total hours spent in patient-facing meetings, documentation, and communication as part of the intervention.

Reviewer 2

The authors have conducted an economic evaluation of a remote person-centred care add-on intervention for persons with chronic heart failure and/or chronic constructive pulmonary disease. The data comes from a randomised controlled trial. The evaluation is competently done and presented in a clear manner.

Major

Participants – please add in text how many of the assessed were eligible as this is the number of interest with regard to participation.	Amendments have been made to both the consort flow chart and the text to more clearly outline the recruitment and screening process. Previously those participants who declined to participate or

	were non-respondents were wrongly classified as not eligible. However, they were invited to participate in the study after the initial inclusion/exclusion criteria screening. The text below has been added to the manuscript.<br><br>**Page 7, Lines 1-6**<br><br>In total 1467 people were assessed for eligibility, with 736 patients not meeting the inclusion/exclusion criteria after the initial screening of medical records. The remaining 731 patients were deemed eligible and posted an information letter inviting them to contact an HCP for further information. Participants who did not contact an HCP after a two-week period were contacted via phone and asked to participate. A total of 224 patients consented to participating in the trial after accounting for nonrespondents (n=237) and those that declined to participate (n=270).<br><br>**Consort Flow Chart Amendment (Figure 1.)**<br><br>**Not eligible (n=736)**<br>Did not meet the inclusion criteria **(n=353)**<br>Excluded **(n=383)**<br>Severe Impairment (n=239)<br>No registered address (n=21)<br>Expected survival <12 months (n=71)<br>Ongoing alcohol or drug abuse (n=33)<br>Diseases that could interfere with follow-up (n=17)<br>Participating in a conflicting study (n=2)<br>**Invited to participate in the study (n=731)**<br>Nonrespondents (n=237)<br>Declined to participate (n=270)<br>Agreed to participate (n=224)
The impact of productivity losses with regard to retirement might impact the results unless the distribution of the two groups is perfectly balanced with regard to age/retirement. It's currently not possible to see this from the presented data (on page 15 it is reported that 89 participants across both groups are above 65 years of age). One way to deal with this would be to include an additional subgroup analysis based on being working aged/retired.	After review, we believe that the paragraph you are indicating was unclear and portrayed separate arguments. The entire paragraph has been re-written to focus on discussing the lack of information with respect to unpaid labour in those who are retired. We also looked more closely at the subgroup population and found that of those who were included over the age of 65, 8 still participated in some form of paid work, with only

	two participants working 75% or more. Text below:<br><br>**Page 16, Lines 25-27 & Page 17, Lines 1-8**<br><br>Health economic evaluations adopt different perspectives when determining the types of health benefits and costs to be included. At its most basic, a societal perspective includes all appropriate healthcare costs, as well as costs associated with productivity loss that arise from a patient's inability to work (30). A societal perspective was applied to this economic evaluation estimating total costs for productivity loss of SEK 4 485 378 in the intervention group and SEK 9 759 578 in the control group. A criticism of this approach is that it often fails to account for the effects of productivity loss in older patients who no longer participate in the labour market, such as unpaid work, including informal caregiving, household production activities and volunteer work (31). Costs related to lost productivity account for approximately 40% of the total healthcare costs for patients with COPD and/or CHF that still participate in the labour market, however, information on the long term effects after retirement are limited (5,32). Within this study stratified sampling, using an estimated retirement age of 65 years, allocated n = 89 participants to each of the intervention and control groups. Within this total population of n=178 participants, eight still participated in the labour market, with only two participants working more than 75%. With such a large number of participants having no calculated productivity loss, there is a risk of under-valuing the true cost-effectiveness of interventions for older patients living with chronic conditions.
I am confused about the inclusion of patient fees. They should not be included in the societal perspective as the full resource utilisation is included in the healthcare costs. However, patient fees are not included in the cost table, so it seems that it is, correctly, excluded. But then,	The addition of patient co-payments aims to provide a descriptive indicator of the out-of-pocket costs incurred by patients. The assumption when conducting the analysis is that co-payments are already included in the price for DRGs and the weighted contacts (although our contacts with the

what is the purpose of the section on patient fees in the method and the last paragraph in the result section? In the "Strengths and limitations", the authors allude to some form of "patient perspective", but this is unclear how this has been analysed. If it is a part of the purpose of the study, it would help the reader if this was clarified, and a part of the aim.	National Board of Health and Welfare and the Swedish Association of Local Authorities and Regions have so far not provided any clear answer to this). Total costs were used for prescribed drugs, meaning the co-payments are also already included. No co-payments were added to these cost categories, thereby avoiding double-counting costs. Below are additions to the manuscript to highlight the costs included in each analysis.<br><br>METHOD **(Page 6, Lines 9-12)**<br>A healthcare payer perspective and patient perspective were also explored to highlight the potential impact of the cost components for the different actors involved in the participation and delivery of healthcare.<br><br>ANALYSIS **(Page 11, Lines 23-26)**<br>In addition to societal costs (total costs for drugs, healthcare costs, and costs for lost productivity), costs were also reported separately from a patient perspective (co-payments for prescribed drugs and healthcare) and healthcare payer perspective (costs for prescribed drugs, and healthcare costs).
The difference in effect is not clearly stated. In the result section, it is presented as HRQoL (0.0469). This is later reported as a QALY difference. Please clarify if this number is the difference in accumulated QALY over the two years or if it is a difference in health state utility. This would potentially be clearer if Table S1 also showed the calculated QALY.	The difference was in accumulated QALY over the two years, and this has been clarified throughout the results section.
Clarify if the costs are significantly different between the groups, or the different cost categories.	Table 2. now includes p-values that show no significant differences between total costs per patient at two years. Amendments have also been made to the results and analysis sections describing this process.

Minor

Title: "Economic evaluation of person-centred care using a digital platform…" is somewhat	We agree that the previous title could be misleading. The intervention is emphasised by

misleading. It is not an evaluation of person-centred care, but a specific support system that aims to achieve person-centred care.	the person-centred care practices accompanied by an **add-on** eHealth support system. Below is an amendment title that we believe states this more clearly.<br><br>"Economic evaluation of a person-centred care intervention with a digital platform and structured telephone support for people with chronic heart failure and/or chronic obstructive pulmonary disease: results from a randomised controlled trial."
A "registry" is where registers are kept. That is, "registry" and "registers" are not interchangeable words.	Corrections have been made to the document.
Add the perspective to your aim	The perspective has been added to the aim<br><br>**Page 5, Lines 25-26**<br><br>The aim of the study was to evaluate the healthcare costs and effects of a remote Person-Centred Care (PCC) add-on intervention compared with usual care for people with Chronic Heart Failure (CHF) and/or Chronic Obstructive Pulmonary Disease (COPD) **from a societal perspective**.
Introduction (p5, line 13): "innovate current methods" – current methods need not be innovated. I guess you mean to "innovate on" or something along those lines.	Following the suggestion the sentence has been changed to "innovate on" as seen below.<br><br>**Page 5, Line 12**<br><br>The burden of these diseases highlights a need to **innovate on** current methods for providing healthcare that promote patient involvement to contain costs and facilitate a transition to more sustainable healthcare systems.
P7 line 6: typo "listed created"	Adjusted to "list created"

"Healthcare costs" explain how the authors have obtained primary care and specialised outpatient care costs when DRG weights are missing. But it does not say how they moved from DRG to costs when it was available. I assume in a manner similar to inpatient costs but would be good if this was clarified.	Upon reflection, the way in which the paragraph is written is confusing. Primary Care data had no DRG weights, as they were not provided for Primary Care in Region Västra Götaland at the time of the data output. Therefore, the process of imputing costs was used for Primary Care and then also for Specialised Outpatient Care when DRG weights were not provided. Both Inpatient and Polyclinical care had complete DRGs and therefore no additional imputation of costs was necessary. I've made amendments to the whole paragraph.<br><br>Please see the response to Reviewer 1 (above). The restructuring of the "Healthcare Costs" paragraph also answers this question.<br><br>**Page 9, Lines 24-26 & Page 10, Lines 1-16**
P 14 line 21: typo "and"	Adjust to "an"
Page 15 line 27: Here "indirect costs" are introduced for the first time. It is unclear what this refers to. Productivity losses?	We believe this has also been addressed in the response to your previous comment about the population group above the age of 65.<br><br>See adjusted text above
Table 2: The intervention cost is presumably the SEK176'492 as presented in the text. But 176492/110=1604.	I have amended the calculation error in Table 2. It now reads 1604.

Suggestions:

Since you have access to healthcare costs, it would be interesting if you also presented the results from the healthcare perspective (payer), as this would be important information for a decision maker and make the results accessible also in contexts where productivity losses are considered inappropriate in economic evaluations. It would also deal with some of the problems you discuss regarding including productivity losses. I do think you are correct in	This has now been added to the manuscript. The intervention group had considerable lower total costs, and the results would follow the same trajectory as the base analysis. The text below was added to the results.<br><br>**Page 15, Lines 6-9**

including productivity losses but excluding them would show how this choice influences the conclusion.	The healthcare payer perspective resulted in total costs of SEK 18 803 977 for the control group and SEK 16 986 442 for the intervention group, following the same trajectory as the societal perspective analysis account only for direct healthcare and prescription drug costs (Table S8).

Additional Modifications:

- A small modification has been made to clarify the limitations of imputations performed within the intervention times. **Page 18, Lines 21-23**

VERSION 2 - REVIEW

Reviewer 2

Name Jarl, Johan

Affiliation Lund University, Health Economics Unit, Department of Clinical Science (Malmö)

Date 28-Aug-2025

COI

Thank you for considering my concerns. I have no further comments.
